# Intravaginal Lactic Acid Bacteria Modulated Local and Systemic Immune Responses and Lowered the Incidence of Uterine Infections in Periparturient Dairy Cows

**DOI:** 10.1371/journal.pone.0124167

**Published:** 2015-04-28

**Authors:** Qilan Deng, John F. Odhiambo, Umar Farooq, Tran Lam, Suzanna M. Dunn, Burim N. Ametaj

**Affiliations:** Department of Agricultural, Food and Nutritional Science, University of Alberta, Edmonton, Alberta, Canada T6G 2P5; Facultad de Medicina, URUGUAY

## Abstract

The objective of this investigation was to evaluate whether intravaginal infusion of a lactic acid bacteria (LAB) cocktail around parturition could influence the immune response, incidence rate of uterine infections, and the overall health status of periparturient dairy cows. One hundred pregnant Holstein dairy cows were assigned to 1 of the 3 experimental groups as follows: 1) one dose of LAB on wk -2 and -1, and one dose of carrier (sterile skim milk) on wk +1 relative to the expected day of parturition (TRT1); 2) one dose of LAB on wk -2, -1, and +1 (TRT2), and 3) one dose of carrier on wk -2, -1, and +1 (CTR). The LAB were a lyophilized culture mixture composed of *Lactobacillus sakei* FUA3089, *Pediococcus acidilactici* FUA3138, and *Pediococcus acidilactici* FUA3140 with a cell count of 10^8^-10^9^ cfu/dose. Blood samples and vaginal mucus were collected once a week from wk -2 to +3 and analyzed for content of serum total immunoglobulin G (IgG), lipopolysaccharide-binding protein (LBP), serum amyloid A (SAA), haptoglobin (Hp), tumor necrosis factor (TNF), interleukin (IL)-1, IL-6, and vaginal mucus secretory IgA (sIgA). Clinical observations including rectal temperature, vaginal discharges, retained placenta, displaced abomasum, and laminitis were monitored from wk -2 to +8 relative to calving. Results showed that intravaginal LAB lowered the incidence of metritis and total uterine infections. Intravaginal LAB also were associated with lower concentrations of systemic LBP, an overall tendency for lower SAA, and greater vaginal mucus sIgA. No differences were observed for serum concentrations of Hp, TNF, IL-1, IL-6 and total IgG among the treatment groups. Administration with LAB had no effect on the incidence rates of other transition cow diseases. Overall intravaginal LAB lowered uterine infections and improved local and systemic immune responses in the treated transition dairy cows.

## Introduction

Dairy cows undergo an immunosuppressive state around parturition associated with impaired leukocyte functions [1,2]. Several investigators have reported that although the phagocytic activity of neutrophils remains high, their bactericidal capacity is weakened, especially after parturition [3–5]. In addition, concentrations of IgG and IgM in the blood reach the lowest concentrations at calving [2]. Moreover, there is lower IgG content in the uterine secretions suggesting a decrease in the local bactericidal activity [6].

The state of immunosuppression in transition cows is associated with high incidence of bacterial infections especially of the uterus (metritis) and mammary gland, rendering cows more vulnerable to periparturient diseases. Almost 40% of periparturient dairy cows are affected by clinical metritis during the first 3 wk after calving and another 15–20% by endometritis more than 3 wk after parturition [7]. Uterine infections predispose dairy cows to impaired reproductive performance and are the number one reason for culling of cows in Canadian dairy herds [8]. Infection of the uterus is accompanied by systemic fluctuations of inflammatory cytokines like tumor necrosis factor (TNF), interleukin (IL)-1, IL-6, and acute phase proteins (APP) such as lipopolysaccharide-binding protein (LBP), serum amyloid A (SAA), and haptoglobin (Hp) [9–11]. Currently there is no efficient treatment for uterine infections. Although various therapies have been used in the past involving antibiotics, iodine solutions, and hormone treatments, for different reasons, they have not been successful enough to be widely embraced by veterinary clinicians [12–14].

On the other hand, a new line of research is growing with the use of probiotic agents as an alternative to antimicrobial compounds. Probiotics are live microorganisms, which confer a health benefit to the host when administered in adequate amounts (World Health Organization/Food and Agricultural Organization, 2001). They have demonstrated the ability to enhance immune functions such as increasing the number of immune cells and modulating expression of cytokines or antibody production in the host [15–17]. Research conducted in human subjects has indicated that probiotics administered in the vagina have been able to lower the incidence of vaginal infections in women [18,19]. However, there is a lack of research in dairy cattle regarding the utilization of probiotics to lower the incidence of uterine infections and improve reproductive performance. In a recent study, we reported that cows treated intravaginally with 2 prepartum and 4 postpartum doses (on a weekly basis) of a mixture of 3 lactic acid bacteria (LAB), isolated from the vaginal tract of healthy pregnant cows, had lower incidence of purulent vaginal discharges and lower concentration of serum Hp than the control animals [20]. In this study, we hypothesized that a lower number of treatments with LAB might confer the same results on uterine health and immune status of transition dairy cows. Therefore, the objectives of this study were to test whether lowering the treatment frequency of intravaginal administration of LAB to 2–3 doses around calving will enhance local and systemic immune responses and lower the incidence of uterine infections and potentially other periparturient diseases of transition dairy cows.

## Materials and Methods

### Ethics Statement

All experimental procedures were approved by the University of Alberta Animal Care and Use Committee for Livestock (Animal use protocol AUP#120), and cows were cared for in accordance with the guidelines of the Canadian Council on Animal Care (1993).

### Animals and Experimental Design

One hundred pregnant Holstein cows were allocated (based on parity, body condition score, and milk yield) to 1 of the 3 experimental groups to receive intravaginal LAB or carrier (sterile skim milk) during the transition period as following: treatment 1 (TRT1)- 2 consecutive LAB doses (on a weekly basis) starting at 2 wk before the expected day of parturition and 1 carrier dose the week after parturition; treatment 2 (TRT2)- 3 consecutive LAB doses (2 doses during the 2 wk before the expected day of parturition and 1 dose the week after parturition); control (CTR)- 3 consecutive carrier doses around parturition starting at 2 wk before the expected day of parturition up to 1 wk after calving. The LAB were composed of *Lactobacillus sakei* FUA3089, *Pediococcus acidilactici* FUA3138 and FUA3140, which were stored in sterile skim milk with a cell count of 10^8^–10^9^ cfu/dose. Both probiotics and carrier were lyophilized and stored at -86°C in vials, and each vial was reconstituted in 1 mL sterile 0.9% saline before administration. The LAB or carrier were infused into the vaginal tract gently with individually wrapped sterile drilled infusion tubes (Continental Plastic Corp., Delavan, WI) capped with a 5-mL sterile screw tip syringe (Becton, Dickinson and Company, Franklin Lakes, NJ), and deposited at cranial vagina. All the procedures were maintained aseptic during administration.

### Clinical Observations and Measurements

All cows were monitored clinically from -2 wk before the expected day of parturition and up to +8 wk after parturition. Rectal temperature was measured twice a week and fever was declared when it was greater than 39.5°C. Retained placenta was declared if a cow did not expel the placenta within 24 hours after parturition. A Sonosite (MicroMaxx, SonoSite, Inc., Bothell, Washington) ultrasound fitted with a 7.5 MHz probe was used on +2, +3, +5, and +7 wk to assess the uterine size and intrauterine fluid. Uterine infections were categorized into different classes. A metritic case was diagnosed if the cow had reddish brown vaginal discharge with fetid odor, together with fever and an abnormally large uterus and decreased feed intake and milk production within 3 wk after parturition. If a cow still had purulent or mucopurulent exudate in the vagina more than 3 wk after parturition, in the absence of systemic illness, she was declared having clinical endometritis. A cow with accumulated purulent materials in the uterine lumen in the presence of a persistent corpus luteum was declared having pyometra [21]. Both clinical endometris and pyometra was monitored until wk +7 postpartum. Total uterine infections were defined as the sum of metritis, clinical endometrtis, and pyometra. Displaced abomasum was diagnosed by a veterinary practitioner based on both clinical signs and the history of the animal. A displaced abomasum was declared by combining the diagnosis result and the veterinary visit records and treatment records of the barn. Lameness was recorded if a cow stood or walked in an abnormal gait, such as reluctance to bear weight on a hoof, or a noticeable limp with uneven steps, especially when she was observed to have a reddish, swollen, hot foot and retracted her foot when touched on the wall of corium. Then the diagnosis of laminitis was conducted by a skilled veterinary practitioner by checking if the corium was swollen or bleeding and also if pulses in the lower limb arteries were prominent. The diagnosed result was then combined with the veterinary visit records and treatment records of the barn to declare a case of laminitis. Subclinical mastitis was declared if somatic cell count (SCC) in milk was more than 200,000 cells/mL [22,23].

### Sampling and Laboratory Analyses

Blood samples were collected from the coccygeal vein once a week in the morning before feeding with 10-mL vacutainer tubes without anticoagulant (BD Vacutainer Systems, Plymouth, UK) from -2 to +3 wk around calving. Blood samples were centrifuged at 2,090 x g and 4°C for 20 min to separate the serum (Beckman Coulter, Pasadena, California). Serum samples were stored at -20°C until analysis. Vaginal mucus was sampled using individually wrapped sterile drilled infusion tubes (Continental Plastic Corp., Delavan, WI) capped with a 5-mL sterile screw tip syringe (Becton, Dickinson and Company, Franklin Lakes, NJ), and then gently flushed into a sterile tube with 1 mL 0.9% saline. All serum and mucus samples were run in duplicate for the lab analyses. Both the inter-assay and the intra-assay coefficients of variation were less than 10%.

Concentrations of lipopolysaccharide binding protein (LBP) in the serum were measured with a commercial sandwich ELISA kit for bovine LBP (Hycult Biotech, Uden, Noord-Brabant, The Netherlands) according to the manufacturer’s instructions. Serum samples were diluted 1:100; then, samples and standards were loaded and incubated to allow LBP to be captured by bovine monoclonal antibodies coated on the plate. After washing, detection antibodies labeled with biotin and horseradish peroxidase (HRP) labeled with streptavidin were loaded to bind the captured LBP. A washing followed each loading. The fixed HRP catalyzed a chromogenic reaction with the subtract 3,3’,5,5’-tetramethylbenzidine (TMB). After adding the stop solution, the plate was read at 450 nm on a microplate spectrophotometer (Spectramax 190, Molecular Devices Corporation, Sunnyvale, CA) within 10 min. The optical density (OD) values were positively correlated with the concentrations of LBP in the sample. The detection range of LBP is between 1.6 and 100 ng/mL. The OD values of samples were within the range of standard curve.

Concentrations of serum amyloid A (SAA) in the serum were measured with a commercial sandwich bovine ELISA kit (Tridelta Development Ltd., Maynooth County Kildare, Ireland) as per manufacturer’s instructions. Serum amyloid A in the samples was captured by both the anti-SAA monoclonal antibodies immobilized on the plate and free anti-SAA monoclonal antibodies labeled with HRP, which catalyzed a chromogenic reaction. The chroma of this enzymatic reaction was proportional to the concentration of SAA in the sample. All serum samples were diluted 1:500 before the assay and the OD values were read on a microplate spectrophotometer (Spectramax 190, Molecular devices Corporation, Sunnyvale, CA) at 450 nm within 10 min after adding the stop solution. The OD values of all diluted samples were within the range of standard curve. According to the manufacturer, the analytical sensitivity of the assay for bovine is 1.5 μg/mL.

Concentrations of haptoglobin (Hp) in the serum were measured with a commercially available kit (Tridelta Development Ltd., Maynooth, County Kildare, Ireland). The assay principle is that the free hemoglobin exhibits peroxidase activity, which is inhibited at a low pH. Haptoglobin binds to hemoglobin and preserves its peroxidase activity at a low pH. The preserved peroxidase activity of hemoglobin is proportional to the amount of Hp in the sample. The OD values of this chromogenic enzymatic reaction were read on a microplate spectrophotometer (Spectramax 190, Molecular devices Corporation, Sunnyvale, CA) at 600 nm 5 min after adding the last reagent. According to the manufacturer, the analytical sensitivity is 0.005 mg/ml.

Concentrations of TNF in the serum were measured with a bovine sandwich ELISA kit (Bethyl Laboratories Inc., Montgomery, TX). Briefly, TNF present in samples and standards is captured by bovine monoclonal antibodies coated on the plate. Then, the captured TNF binds to detection antibodies labeled with HRP. The addition of substrate TMB triggers a chromogenic enzymatic reaction catalyzed by HRP. The color is positively correlated with the concentration of TNF in the sample. The OD values were read at 450 nm with a spectrophotometer (Spectramax 190, Molecular Devices Corporation, Sunnyvale, CA) within 10 min after adding the stop solution. The detection range of TNF is between 0.078 and 5 ng/mL.

Concentrations of interleukin 1 (IL-1) in the serum were measured with a competitive inhibition bovine ELISA kit (Cusabio Biotech Co., Ltd, Wuhan, Hubei, China). Interleukin-1 in the serum samples and standards competed with biotin-conjugated IL-1 to bind IL-1 antibodies coated on the plate. The greater the amount of IL-1 in the sample, the less antibodies bound by biotin-conjugated IL-1. Then, the biotin conjugates avidin is combined with HRP, which catalyzes a chromogenic reaction. The chroma of the color develops in opposite to the amount of IL-1 in the sample. The OD values were measured at 450 nm with a spectrophotometer (Spectramax 190, Molecular Devices Corporation, Sunnyvale, CA) within 10 min after adding the stop solution. No significant cross-reactivity or interference was observed. According to the manufacturer, the minimum detectable dose of bovine IL-1 is less than 250 pg/mL.

Concentrations of IL-6 in the serum were measured with a sandwich ELISA kit for bovine IL-6 (Uscn Life Science Inc., Wuhan, Hubei, China). The principle of the assay is similar with that of TNF. The assay was done with original serum samples without dilution and the concentrations of all samples were within the range of the standard curve. This assay has high sensitivity and specificity for detection of bovine IL-6. According to the manufacturer, the minimum detectable amount of bovine IL-6 is less than 3.3 pg/mL.

Concentrations of total IgG in the serum were measured with bovine IgG (total) ELISA kits (Alpha Diagnostic International Inc., San Antonio, TX). The principle of the assay is similar with that of IL-1. Serum samples were originally diluted 1:100,000 by three dilutions before the assay. The OD values of this chromogenic enzymatic reaction were read on a microplate spectrophotometer (Spectramax 190, Molecular Devices Corporation, Sunnyvale, CA) at 450 nm within 30 min after adding the stop solution. According to the manufacturer, the sensitivity of this assay is 5 ng/ml and has less than 1% cross-reactivity with serum IgG from other animals.

The concentrations of secretory immunoglobulin A (sIgA) in the vaginal mucus were measured with competitive inhibition bovine ELISA kits (Cusabio Biotech Co., Ltd, Wuhan, Hubei, China). The principle of the assay is similar with that of IL-1. Mucus samples were vortexed and centrifuged at 2,090 x g for 20 min (Beckman Coulter, Pasadena, California), and then the supernatant was collected and diluted 1:100 before the assay. According to the manufacturer, the minimum detectable dose of bovine sIgA is less than 2.4 μg/mL. No significant cross-over or interference between bovine sIgA and analogues was observed.

### Statistical Analyses

Data of rectal temperature and serum variables, including concentrations of LBP, SAA, Hp, TNF, IL-1, IL-6, and total IgG, as well as the concentration of sIgA in the vaginal mucus were analyzed using SAS 9.2 software (SAS Institute Inc., Cary, NC). MIXED procedure with repeated measurement was used to test the model as following: Y_ijk_ = μ + T_i_ + W_j_ + (TW)_ij_ +e_ijk_, where μ = the overall population mean; T_i_ = the effect of treatment; W_j_ = the effect of week; (TW)_ij_ = the interaction between treatment and week; and e_ijk_ = residual error. The covariance structure for each variable was modeled separately according to the smallest values of the fit statistics based on the Bayesian information criteria.

Binary data of diseases were analyzed using procedure FREQ with Fisher’s Exact Test to test the effect of treatment. Significance was declared at *P* < 0.05, and tendency at 0.05 ≤ *P* < 0.10.

## Results

### Effect of Intravaginal LAB Treatment on Uterine Infections and Other Periparturient Diseases

The results of clinical observation of periparturient diseases are presented in Table 1. Data showed that LAB treated cows had lower incidence rate of metritis compared with those in the CTR group (*P* < 0.01). TRT1 and TRT2 lowered the incidence rate of metritis by 22% (*P* < 0.05) and 32% (*P* < 0.01), compared with the CTR group, respectively. Although there were no differences in terms of the incidence of clinical endometritis and pyometra, LAB-treated cows had a lower incidence of total uterine infections composed of metritis, clinical endometritis, and pyometra, compared with the CTR cows (*P* = 0.01). Both TRT1 and TRT2 had a numerically lower incidence of retained placenta than CTR cows (3% in TRT1, 6% in TRT2, and 13% in CTR cows). During our experimental period, no cases of displaced abomasum were diagnosed out of 66 LAB-treated cows, but 2 out of 32 cows (6%) in the CTR group. However, this difference did not reach a significant level. There was no difference in the incidence rate of subclinical mastitis indicated by a SCC greater than 200,000 cells/mL. No difference was observed also regarding the incidence rates of laminitis among the treatment groups.

**Table 1:** Effect of LAB on periparturient diseases of transition dairy cows.

	TRT1^1^	TRT2^2^	CTR^3^	*P*
**Metritis, % (case/total)**	15 (5/34)^b^	6 (2/32)^b^	38 (12/32)^a^	0.007
**Clinical endometritis, % (case/total)**	6 (2/34)	9 (3/32)	13 (4/32)	0.62
**Pyometra, % (case/total)**	3 (1/34)	6 (2/32)	3 (1/32)	0.84
**Total uterine infections, % (case/total)** ^4^	24 (8/34)^b^	22 (7/32)^b^	53 (17/32)^a^	0.01
**Retained placenta, % (case/total)**	3 (1/34)	6 (2/32)	13 (4/32)	0.29
**Displaced abomasum, % (case/total)**	0 (0/34)	0 (0/32)	6 (2/32)	0.33
**Subclinical mastitis, % (case/total)** ^5^	40 (8/20)	50 (10/20)	55 (11/20)	0.90
**Laminitis, % (case/total)**	9 (3/34)	9 (3/32)	6 (2/32)	1.00

^1^ TRT1: two prepartum doses of LAB.

^2^ TRT2: two prepartum doses plus one postpartum dose of LAB.

^3^ CTR: carrier only.

^4^ Total uterine infections comprised metritis, clinical endometritis and pyometra.

^5^ Subclinical mastitis was declared when somatic cell count in milk was greater than 200,000/mL within five weeks after parturition.

^a-b^Numbers within a row with different superscript letters are different at *P* < 0.05.

**Fig 1:**
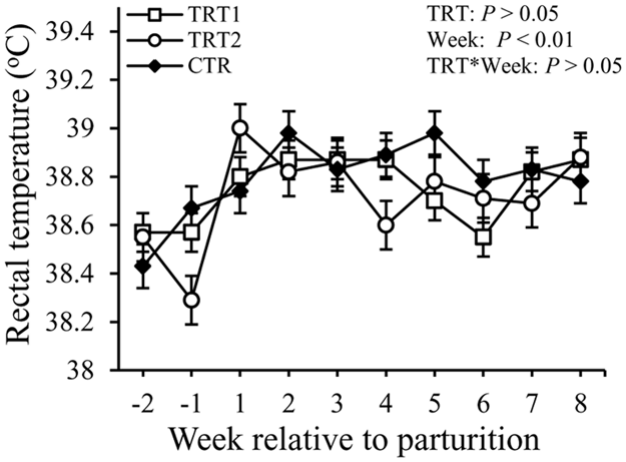
Effect of treatment on the rectal temperature of dairy cows. TRT = effect of treatment; Week = effect of week; TRT * Week = effect of the interaction between treatment and week. TRT1: □, two prepartum doses of LAB, n_1_ = 34; TRT2: ○, two prepartum doses plus one postpartum dose of LAB, n_2_ = 32; CTR:♦, carrier only, n_3_ = 32. LSM ± SEM.

Overall there was an increase of approximately 0.3°C in the rectal temperature of dairy cows after calving compared with pre-calving (*P* < 0.01, Fig 1). However, the rectal temperature was not affected by the treatment and or the interaction between treatment and week.

### Vaginal Immune Responses to Intravaginal LAB Treatment

**Fig 2:**
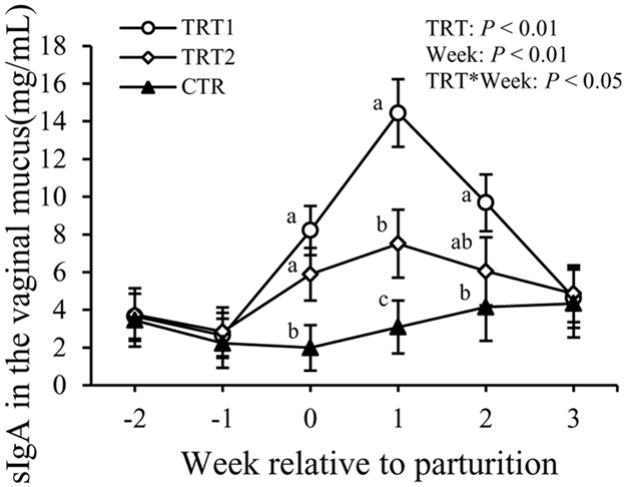
Effect of treatment on the concentration of sIgA in the vaginal mucus. sIgA: secretory immunoglobulin A. TRT = effect of treatment; Week = effect of week; TRT * Week = effect of the interaction between treatment and week. TRT1: □, two prepartum doses of LAB; TRT2: ○, two prepartum doses plus one postpartum dose of LAB; CTR: ♦, carrier only. LSM ± SEM, n = 10 in each group.

Concentrations of sIgA in the vaginal mucus were affected by LAB treatment (*P* < 0.01), week (*P* < 0.01), and the interaction between treatment and week (*P* < 0.01, Fig 2). At wk 0, cows in both TRT1 and TRT2 had greater concentrations of sIgA than cows in the CTR group, but no difference between TRT1 and TRT2 was evidenced. At wk +1, TRT1 had greater concentrations of sIgA than both TRT2 and CTR (*P* < 0.01), whereas cows in the TRT2 had greater sIgA in the vaginal mucus compared to those in the CTR group (*P* < 0.01). At wk +2, TRT1 had greater concentrations of sIgA than both the TRT2 and CTR groups (*P* < 0.01). However, no differences were detected among treatment groups at wk -1 and +3.

### Systemic Immune Responses to Intravaginal LAB Treatment

**Fig 3:**
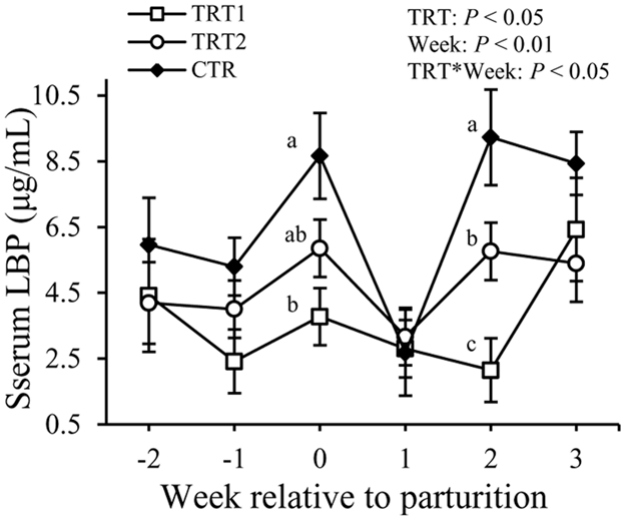
Effect of treatment on the concentration of LBP in the serum. LBP: lipopolysaccharide binding protein. TRT = effect of treatment; Week = effect of week; TRT * Week = effect of the interaction between treatment and week. TRT1: □, two prepartum doses of LAB; TRT2: ○, two prepartum doses plus one postpartum dose of LAB; CTR: ♦, carrier only. LSM ± SEM, n = 10 in each group.

Concentrations of LBP in the serum varied with both treatment (*P* < 0.05) and week (*P* < 0.01, Fig 3). Also, there was an interaction between treatment and week (*P* < 0.05). At wk 0, TRT1 had lower (3.8 ± 0.8 vs. 8.7 ± 1.3 μg/mL, *P* < 0.01), and TRT2 tended to have lower (5.9 ± 0.8 vs. 8.7 ± 1.3 μg/mL, *P* = 0.08) concentrations of LBP in the serum compared with the CTR cows. At wk +2, cows in both TRT1 and TRT2 had lower concentrations of LBP relative to those in the CTR group (2.1 ± 0.9, 5.8 ± 0.8, and 9.2 ± 1.4 μg/mL in TRT1, TRT2, and CTR, respectively, *P* < 0.05).

**Fig 4:**
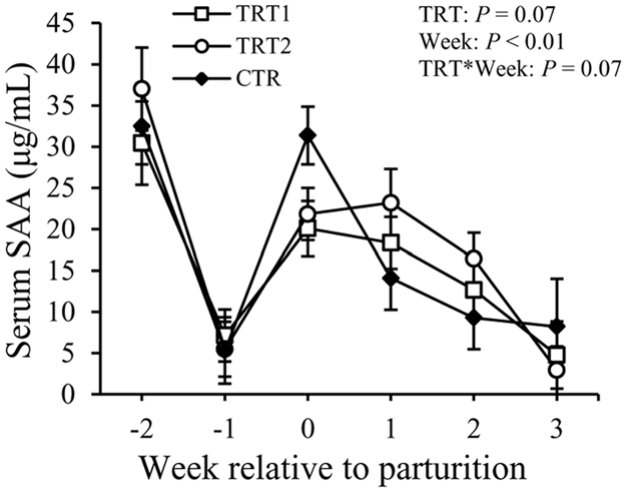
Effect of treatment on the concentration of SAA in the serum. SAA: serum amyloid A; TRT = effect of treatment; Week = effect of week; TRT * Week = effect of the interaction between treatment and week. TRT1: □, two prepartum doses of LAB; TRT2: ○, two prepartum doses plus one postpartum dose of LAB; CTR: ♦, carrier only. LSM ± SEM, n = 10 in each group.

Concentrations of SAA in the serum tended to differ among the treatment groups (*P* = 0.07, Fig 4). Week also had an impact on SAA (*P* < 0.01). Moreover treatment tended to interact with week (*P* = 0.07). At wk 0, cows in the TRT1 tended to have lower concentrations of SAA in the serum compared to those in the CTR group (20.1 ± 3.3 vs. 31.4 ± 5.0 μg/mL, *P* = 0.06). No difference was detected among treatment groups at other weeks. Serum SAA declined at wk -1, followed by an increase from wk 0 to wk +2, and then dropped to a level comparable to wk -1 (*P* < 0.01).

**Fig 5:**
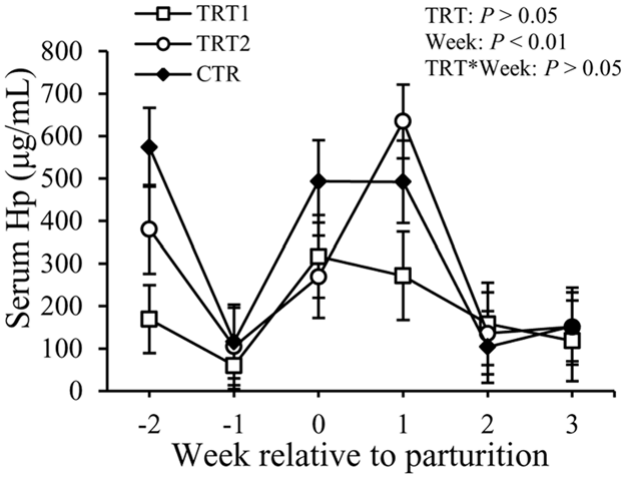
Effect of treatment on the concentration of Hp in the serum. Hp: haptoglobin; TRT = effect of treatment; Week = effect of week; TRT * Week = effect of the interaction between treatment and week. TRT1: □, two prepartum doses of LAB; TRT2: ○, two prepartum doses plus one postpartum dose of LAB; CTR: ♦, carrier only. LSM ± SEM, n = 10 in each group.

Concentrations of Hp in the serum fluctuated with week (*P* < 0.01), but not among the treatment groups or the interaction between week and treatment (Fig 5). Concentrations of Hp in the serum were 182 ± 64 μg/mL, 279 ± 52 μg/mL, and 317 ± 67 μg/mL in TRT1, TRT2, and CTR groups, respectively, (*P* > 0.05).

**Fig 6:**
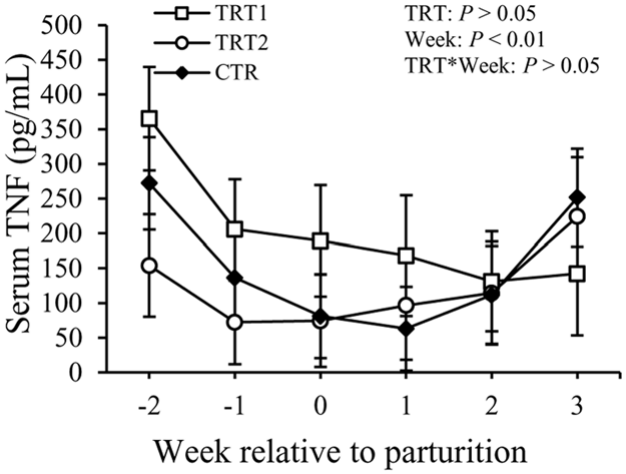
Effect of treatment on the concentration of TNF in the serum. TNF: tumor necrosis factor-α; TRT = effect of treatment; Week = effect of week; TRT * Week = effect of the interaction between treatment and week. TRT1: □, two prepartum doses of LAB; TRT2: ○, two prepartum doses plus one postpartum dose of LAB; CTR: ♦, carrier only. LSM ± SEM, n = 10 in each group.

**Fig 7:**
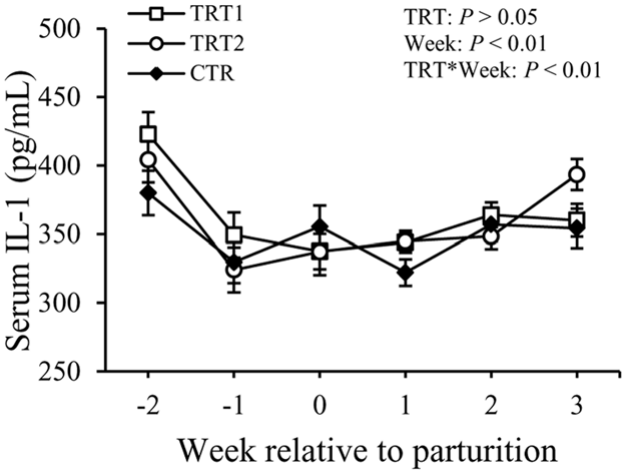
Effect of treatment on the concentration of IL-1 in the serum. IL-1: interleukin-1; TRT = effect of treatment; Week = effect of week; TRT * Week = effect of the interaction between treatment and week. TRT1: □, two prepartum doses of LAB; TRT2: ○, two prepartum doses plus one postpartum dose of LAB; CTR: ♦, carrier only. LSM ± SEM, n = 10 in each group.

**Fig 8:**
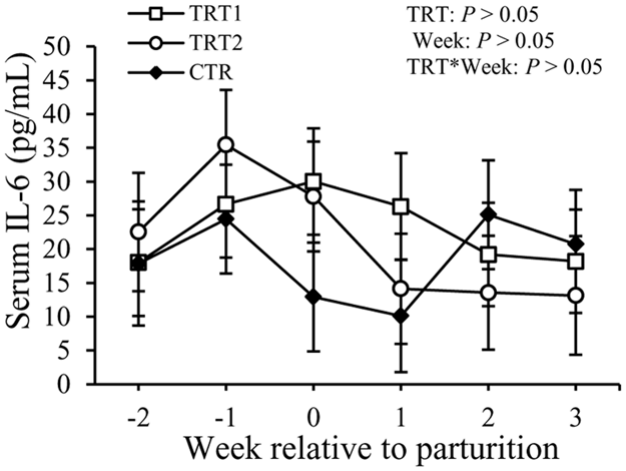
Effect of treatment on the concentration of IL-6 in the serum. IL-6: interleukin-6; TRT = effect of treatment; Week = effect of week; TRT * Week = effect of the interaction between treatment and week. TRT1: □, two prepartum doses of LAB; TRT2: ○, two prepartum doses plus one postpartum dose of LAB; CTR: ♦, carrier only. LSM ± SEM, n = 10 in each group.

Concentrations of TNF in the serum showed a difference with the factor week (*P* < 0.05) but not with the treatment (Fig 6). Concentrations of TNF were 200 ± 64 pg/mL in TRT1, 123 ± 60 pg/mL in TRT2, and 153 ± 84 pg/mL in the CTR group (*P* > 0.05). Concentrations of IL-1 in the serum did not exhibit differences among the treatment groups, but varied with week (*P* < 0.01, Fig 7). There also was an interaction between treatment and week (*P* < 0.01). No differences among treatment groups or in relation with week were observed regarding the concentration of IL-6 in the serum (Fig 8). Overall, concentrations of IL-6 in the serum were 23 ± 9.6 pg/mL in TRT1, 22 ± 9.9 pg/mL in TRT2, and 18 ± 11.4 pg/mL in CTR (*P* > 0.05).

**Fig 9:**
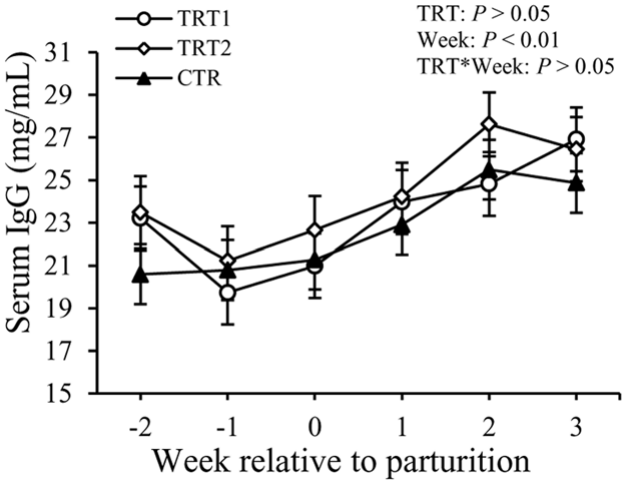
Effect of treatment on the concentration of total IgG in the serum. IgG: immunoglobulin G. TRT = effect of treatment; Week = effect of week; TRT * Week = effect of the interaction between treatment and week. TRT1: □, two prepartum doses of LAB; TRT2: ○, two prepartum doses plus one postpartum dose of LAB; CTR: ♦, carrier only. LSM ± SEM, n = 10 in each group.

Concentrations of total IgG in the serum did not show differences among treatment groups, but varied with week (*P* < 0.01, Fig 9). No significant effect with regards to interaction between treatment and week was observed. The concentrations of IgG were 24 ± 0.9 mg/mL, 24 ± 1.1 mg/mL, and 23 ± 0.9 mg/mL in TRT1, TRT2, and CTR, respectively. Concentrations of IgG in the serum decreased slightly at wk -1, and then gradually increased after calving (*P* < 0.01).

## Discussion

This study was conducted to test the hypothesis that intravaginal administration of LAB around calving can lower the incidence of uterine infections, modulate local and systemic immune responses, and improve the overall health of transition dairy cows. Indeed data showed that cows treated with LAB had lower incidence rates of metritis and total uterine infections and improved systemic and local innate immune responses. However, intravaginally supplemented LAB, at the dose and frequency used in this study, had no effect on the incidence of other periparturient diseases.

One of the most important finding of this study was that the incidence of metritis and total uterine infections were lowered by administration of LAB in the treated cows. Of note, in this study the uterine size was measured by both rectal palpation and ultrasonic imaging. Ultrasonic imaging also was used to monitor the intrauterine fluid and the exudate in the vaginal tract. In addition, rectal temperature and vaginal discharges were taken and observed twice a week to assist diagnosis of uterine infections until 7-wk postpartum. The lower incidence rates of metritis and total uterine infections obtained in this study confirmed our previous finding that intravaginal LAB lowers purulent vaginal discharges in the treated cows [20]. *Lactobacillus* spp., such as *L*. *rhamnosus* GG, *L*. *rhamnosus* GR-1, *L*. *fermentum* RC-14, and *L*. *acidophilus* are well-known for their ability to maintain and restore a normal vaginal microflora and therefore have been used to prevent and treat urogenital infections in women [24–26]. Other than the oral route, *Lactobacillus* spp. has been administered directly in the vagina attenuating or treating symptoms of vaginal infections [27,28]. The results of this study imply that intravaginal administration of LAB confers a health benefit to the reproductive tract against bacterial infections of dairy cows.

Another important finding of this study was that infusion of LAB in the vaginal tract of cows increased concentrations of sIgA in the vaginal mucus. Secretory IgA is recognized as the most important mucosal immunoglobulin of mucosal tissues. The mechanism by which LAB increased sIgA is not fully understood; however, there are reports demonstrating that commensal bacteria can stimulate the production of sIgA with the involvement of local epithelial cells and dendritic cells (DCs) [29]. A great proportion of sIgA in the vaginal mucus originates from local production and not from plasma [30]. The LAB infused in the vagina of the treated cows in our experiment are commensal bacteria identified and isolated from healthy vaginal tracts of cows at our dairy farm as previously described by Wang et al. (2013) [31] and Ametaj et al. (2014) [20]. Usually the host recognizes pathogen-associated molecular patterns (PAMP) like LPS, lipoproteins, peptidoglycans, other polysaccharides, and repetitive protein structures from pathogenic bacteria as ‘danger signals’ and responds by various immune mechanisms including production of specific sIgA [32]. The major function of sIgA is to control the adhesion and uptake of mucosal organisms including commensal bacteria. Therefore, the increased secretion of sIgA in the vaginal mucus induced by administration of LAB can be regarded as strengthening of barrier functions in the vagina. An interesting feature of sIgA is that they are non-inflammatory because they prevent bacterial invasion and colonization by forming immune aggregation and have no complement-activating capability [33,34]. In addition, Boullier et al. (2009) found that sIgA was able to dampen the inflammation at mucosal tissues [33]. The LAB-treated cows had greater concentrations of sIgA in the vaginal tract on wk 0 (immediately after calving), +1 and +2. This was probably due to the invasion of massive pathogenic bacteria into the reproductive tract during this period, as Kaila et al. (1992) found that *Lactobacillus* could promote the development of sIgA specific-antibody producing cells and therefore enhance the secretion of local sIgA in the presence of pathogenic bacteria [35].

Another important finding of this study was that concentrations of LBP in the serum were lowered by intravaginal administration of LAB. One of the main functions of LBP is to facilitate the transfer of LPS to macrophages or lipoproteins so that it could be cleared from the systemic circulation [36,37]. Lipopolysaccharide binding protein is a positive APP elevated in case of inflammatory conditions in ruminants [38]. Bacterial infections of the uterus are commonly present in the postpartum dairy cows, which are associated with histological lesions and inflammation of the uterine tissue [21]. In the first week postpartum, the inflammation is usually confined within the uterus. However, if the local immune responses are not able to resolve the infection, which is usually the case, bacterial endotoxins and locally produced inflammatory cytokines are absorbed into the blood circulation [39–41], triggering systemic inflammatory responses as indicated by elevated concentrations of APP and cytokines in the blood [9,42]. The reason for lower concentrations of LBP might be related to the beneficial effects of LAB on improving mucosal immunity of the reproductive tract and lowering of the amount of LPS entering the systemic circulation, hence lessened systemic inflammation.

Meanwhile, concentrations of serum SAA tended to be lower in LAB treated cows; however, the difference did not reach significance. It is important to note that concentrations of SAA in the serum of LAB-treated cows were lower compared to CTR cows on wk 0 (week of parturition). Serum amyloid A is another APP produced by hepatocytes which can increase up to 1000-fold in the blood within hours following infections [43]. Meanwhile, it is also an apolipoprotein associated with high-density lipoproteins (HDL). High-density lipoproteins exhibit a high binding ability to LPS due to their elevated phospholipid content [44]. Therefore, in the presence of LPS, SAA-containing HDL binds to LPS forming a complex, and then removing it from circulation and disposing it in tissues that express lipoprotein receptor, mainly in the liver [45–47]. Consequently, LPS is removed from hepatocytes through excretion into the bile [48]. The tendency for a lower concentration of SAA in the serum of treated cows is another evidence of a lessened systemic inflammation in the LAB-treated cows.

Haptoglobin is another acute phase protein known for its binding to hemoglobin and preventing iron utilization by bacteria. Although Hp is considered a sensitive APP for bacterial infections of the uterus in ruminants [20,38], the concentrations of Hp in the serum were not different between the LAB treated groups and the CTR group. No differences were detected among treatment groups in terms of concentrations of TNF, IL-1, and IL-6 in the serum of cows. Concentrations of total IgG in the serum also were not affected by the LAB treatment in this study. Moreover, administration of LAB in the vaginal tract of transition cows did not have an effect on the incidence rates of other periparturient diseases like retained placenta, displaced abomasum, subclinical mastitis, and laminitis.

## Conclusions

The results of this study indicated that intravaginal administration of LAB lowered the incidence of uterine diseases in the treated cows and altered their mucosal and systemic immune responses. Lowered incidence rates of metritis and total uterine infections of postpartum dairy cows were associated with enhanced sIgA production in the vaginal mucus. Cows administered intravaginally with LAB had lower systemic inflammation as denoted by lower concentrations of LBP and a tendency of lower SAA in the serum of the treated cows. It can be concluded that intravaginal administration of LAB holds the potential to improve immune status and lower the risk of uterine infections of transition dairy cows.
